# Risk of sexual transmission of HIV in the context of viral load suppression

**DOI:** 10.14745/ccdr.v49i1112a01

**Published:** 2023-11-01

**Authors:** Pascal Djiadeu, Housne Begum, Stacy Sabourin, Stephan Gadient, Chris Archibald, Marc-André LeBlanc, Andrea Chittle, Annie Fleurant, Joseph Cox

**Affiliations:** 1 Centre for Communicable Diseases and Infection Control, Public Health Agency of Canada, Ottawa, ON

**Keywords:** HIV, viral load, sexual transmission, serodiscordance, viral suppression, gbMSM

## Abstract

**Background:**

In 2018, the Public Health Agency of Canada (PHAC) published a systematic review to calculate the risk of sexual transmission of human immunodeficiency virus (HIV) in the context of antiretroviral therapy (ART). In 2022, PHAC commissioned the Canadian Agency for Drugs and Technologies in Health (CADTH) to conduct a rapid review of evidence published since 2017. We undertook a meta-analysis of relevant studies from these two reviews.

**Methods:**

Studies from the rapid review that adequately assessed exposure (HIV viral load) and outcome (HIV seroconversion) were included and assessed for risk of bias (RoB) and certainty of evidence. Results were pooled to estimate the risk of HIV transmission per 100 person-years.

**Results:**

Three studies from the rapid review were eligible for inclusion and one was excluded after RoB assessment. In the remaining studies examining risk among people living with HIV who take ART and maintain a suppressed viral load (fewer than 200 copies/mL, measured every 4–6 months), no sexual transmissions of HIV were observed. The pooled incidence estimate based on these studies, and one from the 2018 PHAC review, was zero transmissions/100 person-years (95% CI: 0.00–0.10). No studies in the rapid review provided data on the risk of sexual transmission of HIV in situations of varying levels of viral load.

**Conclusion:**

This update highlights the consistency of evidence since the 2018 PHAC review. There remains no evidence of HIV transmission to sexual partners when a person living with HIV is on ART and maintains a suppressed viral load.

## Introduction

Human immunodeficiency virus (HIV) is a retrovirus that progressively destroys CD 4+ lymphocytes, which are crucial to immune system functioning. If not treated, HIV can progress to acquired immunodeficiency syndrome (AIDS). Human immunodeficiency virus can be transmitted through exposure to blood, semen, vaginal fluid, rectal fluid and human milk ((1,2)). In Canada, the annual number of new diagnosed cases of HIV infection has remained relatively stable since 2012, with 1,472 cases reported in 2021 ((3,4)). As of 2020, an estimated 90% of persons living with HIV in Canada had been diagnosed and were aware of their infection. Of those diagnosed, 87% were estimated to be on treatment, and an estimated 95% of persons on treatment had a suppressed viral load of fewer than 200 copies/mL ((4)). Viral load is the measure of the amount of HIV ribonucleic acid circulating in the blood. In 2020, it was estimated that 77% of new HIV infections occurred through sexual transmission ((4)). Among people living with HIV, higher viral load levels are associated with increased risk of sexual transmission of HIV ((5–8)).

In 2018, the Public Health Agency of Canada (PHAC) published a systematic review to calculate the risk of sexual transmission of HIV ((9)). The 2018 PHAC review found that the overall risk of sexual transmission of HIV when the partner living with HIV was taking antiretroviral therapy (ART) with varying levels of viral load was 0.22 transmissions per 100 person-years (PY) (pooled 95% confidence interval [CI]: 0.14–0.33), across heterosexual and gay, bisexual and other men who have sex with men (gbMSM) serodiscordant couples. Furthermore, the review determined that the overall risk when a person living with HIV was taking ART and had a suppressed viral load (defined as fewer than 200 copies/mL measured every 4–6 months) was zero transmissions per 100 PY (pooled 95% CI: 0.00–0.28).

In 2022, PHAC commissioned the Canadian Agency for Drugs and Technologies in Health (CADTH) to carry out a rapid review of new evidence published since the 2018 PHAC review. The CADTH rapid review focussed on the risk of sexual transmission of HIV when a person living with HIV is taking ART (with varying levels of viral load) or is taking ART and has a suppressed viral load ((10)).

The CADTH rapid review identified 15 studies published between 2017 and 2022 that were relevant to the research questions, including one systematic review and 14 non-randomized studies ((10)). The rapid review did not evaluate the certainty of the evidence of each study, but rather described their strengths and limitations narratively. This rapid communication includes further analyses of studies included in the CADTH rapid review and provides an updated risk of sexual transmission of HIV when a person living with HIV is taking ART.

## Methods

Relevant studies from the CADTH rapid review were first identified based on the use of valid measures of exposure (viral load testing) and outcome (phylogenetic linkage of observed seroconversions to the partner living with HIV). Included studies were further evaluated for risk of bias (RoB) and certainty of evidence using the Quality in Prognosis Studies instrument and Grading of Recommendations Assessment, Development, and Evaluation (GRADE) criteria, respectively ((11,12)). Results from retained studies were pooled using a random-effects model to calculate pooled estimates of the risk of HIV transmission per 100 PY with 95% CIs. Analyses were done using R studio with the meta package: Meta-Analysis Package (v2.4-0) ((13,14)).

As in the 2018 PHAC review, HIV transmission risk was characterized using criteria defined by the Canadian AIDS Society (**Appendix**, **Table A1**) ((15)).

## Results

Risk of bias and certainty of evidence of studies included in the CADTH rapid review

Regarding the risk of sexual transmission of HIV when a person living with HIV takes ART (with varying levels of viral load), only two studies were of potential relevance (Appendix, **Table A2**) ((16,17)).

The article by Nyombayire *et al.*, ((16)) had methodologic limitations, including a high RoB (Appendix, **Table A3**) and a very low certainty of evidence (Appendix, **Table A4**). The article by Bavinton *et al.*, ((17)) found no phylogenetically linked HIV transmissions when the partner living with HIV had varying levels of viral load and the partner did not use HIV pre-exposure prophylaxis (PrEP), but the article had only 5.8 PY of relevant follow-up. The certainty of evidence in this article was evaluated as very low (Appendix, **Table A5**). The RoB was high due to the lack of information on those who chose not to participate in the study, limited viral load reporting, no validation of ART adherence and considerable loss to follow up. In addition, not all reported transmissions were phylogenetically linked to the partner living with HIV. Given the above stated limitations, neither article was considered to add meaningful information to the 2018 PHAC review conclusions for this question.

Regarding the risk of sexual transmission of HIV when a person living with HIV takes ART and has a suppressed viral load (fewer than 200 copies/mL measured every 4–6 months), the CADTH rapid review found two observational studies among gbMSM (Table A2) that met the inclusion criteria, both of which were follow-up studies to work previously included in the 2018 PHAC review ((17,18)). The RoB was evaluated as moderate for the article by Bavinton *et al.*, ((17)) and low for Rodger *et al.*, ((18)) (Table A3), while the certainty of evidence on this question for both studies was evaluated as high (Table A5).

### Public Health Agency of Canada analysis and pooled risk of sexual transmission of eligible studies

**Figure A1:**
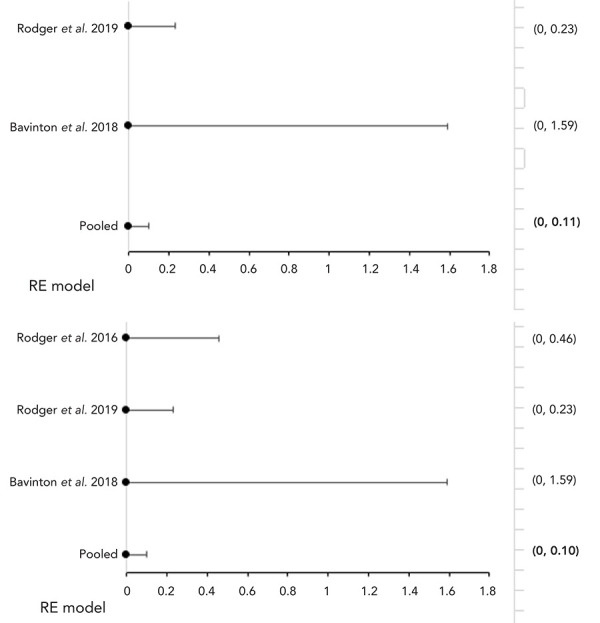
Pooled estimate of the risk of HIV transmission per 100 person-years among gbMSM and heterosexual serodiscordant couples^a,b^ Abbreviations: ART, antiretroviral therapy; CI, confidence interval; gbMSM, gay, bisexual and other men who have sex with men; HIV, human immunodeficiency virus; RE, random effect ^a^ References Nyombayire *et al.* ((16)) and Bavinton *et al.* ((17)) ^b^ The pooled estimate includes heterosexual partners from Rodger *et al.* ((19)) and gbMSM partners from Rodger *et al.* ((18)) and Bavinton *et al.* ((17))

Two studies provided additional evidence regarding the risk of sexual transmission of HIV for gbMSM couples when the person living with HIV has a suppressed viral load. In these studies, no sexual transmissions of HIV that were phylogenetically linked were reported ((17,18)). The estimated incidence was zero transmissions/100 PY (95% CI: 0.00–0.23) for the article by Rodger *et al.*, ((18)) and zero transmissions/100 PY (95% CI: 0.00–1.59) for the article by Bavinton *et al.*, ((17)). Data from these studies were pooled to estimate an incidence of zero transmissions/100 PY (95% CI: 0.00–0.11) (Appendix, **Figure A1**).

The 2018 PHAC review included only one article ((19)) that provided data on the risk of HIV transmission for heterosexual couples where the partner living with HIV has a suppressed viral load. The estimated incidence was zero transmissions/100 PY (95% CI: 0.00–0.46) ((9,19)). No articles in the CADTH rapid review provided additional data for this population.

To update the 2018 PHAC review results for a combined (heterosexual and gbMSM) estimate of the risk of sexual transmission when a person living with HIV has a suppressed viral load, we pooled the results of Bavinton *et al.*, ((17)) and Rodger *et al.*, ((18,19)). This resulted in an incidence estimate of zero transmissions/100 PY (95% CI: 0.00–0.10) (Figure A1). With additional data, there is more precision around the estimated incidence, so that the 95% CI of 0.00 to 0.28 documented in the 2018 PHAC review ((9)) is now 0.00 to 0.10.

## Discussion

The 2023 PHAC analysis of relevant studies from the CADTH rapid review did not provide any new evidence to alter the conclusions from the 2018 PHAC review related to the risk of sexual transmission of HIV when a person living with HIV takes ART (with varying levels of viral load). Therefore, the risk of HIV transmission in this situation remains categorized as low, as per Canadian AIDS Society guidelines (Table A1). Future work is needed to determine more precise transmission risk estimates for situations involving varying levels of viral load.

Regarding the risk of sexual transmission of HIV when a person living with HIV takes ART and has a suppressed viral load of fewer than 200 copies/mL measured every 4–6 months, the CADTH rapid review found two updated studies among gbMSM. These studies, in addition to a single study on heterosexual couples, identified in the 2018 PHAC review, allowed an update of the meta-analysis from the 2018 PHAC review, resulting in more precision for the estimated risk of sexual transmission (zero transmissions/100 PY; 95% CI: 0.00–0.10). This updated review offers additional support to the conclusions of the 2018 PHAC review, further documenting no confirmed cases of sexual HIV transmission when a person living with HIV maintains a suppressed viral load. The risk of HIV transmission in this situation remains categorized as negligible, as per Canadian AIDS Society guidelines (Table A1). Communicating this message has the potential to reduce HIV-associated stigma and support increased engagement across the HIV care continuum, with benefits for individuals and communities.

## Conclusion

This meta-analysis of updated articles derived a more precise estimate of the risk of sexual transmission of HIV when a person living with HIV is taking ART and maintains a suppressed viral load (fewer than 200 copies/mL, measured every 4–6 months). With five years of additional data, the conclusion of the 2018 PHAC review is strengthened. There remains no evidence of HIV transmission to sexual partners when a person living with HIV is on ART and maintains a suppressed viral load.

## Appendix

**Table A1:** Categories for assessing HIV transmission risk^a^

Category	Description	Criteria for determining level of risk	
No risk	None of the practices in this category have ever been demonstrated to lead to HIV infection. There is no potential for transmission since all the basic conditions for viral transmission are not present.	Potential for transmission	None
		Evidence of transmission	None
Negligible risk	All the practices assigned to this risk level present a potential for HIV transmission because they involve an exchange of bodily fluids (semen, pre-ejaculate, rectal fluid, vaginal fluid, blood, or breast milk). However, the amounts, conditions and media of exchange are such that the efficiency of HIV transmission appears to be greatly diminished. There are no confirmed reports of infection from these activities.	Potential for transmission	Yes
		Evidence of transmission	None
Low risk	All of the practices assigned this risk level present a potential for HIV transmission because they involve an exchange of bodily fluids (semen, pre-ejaculate, rectal fluid, vaginal fluid, blood, or breast milk). There are also a few reports of infection attributed to these activities (usually through individual case studies or anecdotal reports, and usually under certain identifiable conditions).	Potential for transmission	Yes
		Evidence of transmission	Yes (under certain conditions)
High risk	All of the practices assigned this risk level present a potential for HIV transmission because they involve an exchange of bodily fluids (semen, pre-ejaculate, rectal fluid, vaginal fluid, blood, or breast milk). In addition, a significant number of scientific studies have repeatedly associated the activities with HIV infection. Even when the exact mechanism of transmission is not completely clear, the results of such studies conclude that activities in this category are high risk.	Potential for transmission	Yes
		Evidence of transmission	Yes

Abbreviation: HIV, human immunodeficiency virus

^a^ Adapted from the Canadian AIDS Society ((15))

**Table A2:** Characteristics of new studies that align with questions of interest in this review^a^

Study, year, country	Study design, setting and period	Population characteristics	Exposure and comparator	Clinical outcome
Bavinton *et al.*, 2018 ((17)) Australia, Brazil, Thailand	Prospective cohort study Setting: 13 Australian clinics; 1 Brazilian clinic; 1 Thai clinic (no other information reported) Study period: May 2012–March 2016	HIV serodiscordant male same-sex couples/sex partners Number of participant couples, n=343 Baseline characteristics (sex partner LWH): Age, median (IQR), years 34.4 (27,7, 43.9) Sex with outside partner(s), n (%): Any=136 (40%) CLAI=59 (17%) Viral load (measure NR) in the sex partner LWH, copies/mL, n (%): <200=267 (78%) ≥200=76 (22%) Daily PrEP use by the HIV-negative partner in the past 3 months, n (%): 26 (8%) ART use at baseline in sex partner LWH, n (%): 274 (80%) ≥90% adherence to ART in the past 3 months at baseline (among 274 sex partners LWH and taking ART), n (%): 241 (88%) Condom use/CLAI in the past 3 months, n (%): Always condoms/no CLAI=156 (45%) Some condoms/CLAI=126 (37%) Always CLAI=61 (18%) Any STI, n (%): Sex partner LWH=46 (13%) HIV-negative partner=39 (11%)	Exposure: Sex partners LWH were virally suppressed (most of whom were using ART) ART regimens: NR ART use in sex partner LWH during the follow-up, n (%): Never=6 (2%) Initiated during follow-up=85 (25%) Always=252 (73%) Viral load in sex partner LWH during the follow-up, n (%): Consistently <200 copies/mL=258 (75%) Variably >/<200 copies/mL=78 (23%) Consistently ≥200 copies/mL=7 (2%) Daily PrEP use by the HIV-negative partner anytime during the follow-up, n (%): 115 (34%) Comparator: None	Outcomes: Primary HIV seroconversion in the HIV-negative partner with viral load monitoring and phylogenetic linkage demonstrated Follow-up: At least 2 clinic visits per year Viral load monitoring was every 3–6 months Total couple years of follow-up=588.4 232 person-year (with suppressed viral load and no PrEP) 5.8 person-year (with varying viral load and no PrEP) Median follow-up/couple (IQR)=1.7 (0.9, 2.2)
Rodger *et al.*, 2019 (18)) PARTNER2 UK (14 European countries)	Single arm prospective cohort study Setting: 75 sites across 14 European countries Study period: 2010–2017	Gay male HIV-serodiscordant couples Inclusion criteria: both partners were ≥18 years of age, had penetrative sex with or without condoms in the month prior to enrolment, expected to have sex together again after enrolment, consent of both partners obtained Exclusion criteria (for analysis): HIV negative partner using HIV PEP or PrEP, reported no condomless sex, viral load of the sex partner LWH >200 copies/mL, absence of viral load data, absence of HIV testing in the HIV negative partner Number of participants, n=782 couples (340 of whom were from PARTNER1) ((19)) Age, median (IQR), years: sex partner LWH=40.0 (33.3, 46.1) HIV negative partner=37.6 (30.9, 45.3) CD 4 cell count in the sex partner LWH, n (%): >350 cells/µL, n=730 (93%) ≤350 cells/µL, n=51 (7%) Number of participants with STIs, n (%): Sex partner LWH=214 (27%) HIV negative partner=185 (24%)	Exposure: Sex partner LWH takes suppressive ART and has viral load <200 copies/mL ART regimen: NR ART in sex partner LWH: Years on ART, median (IQR)=4.3 (1.8, 9.3) Self-reported ART adherence, n (%): ≥90%=739 (98%) <90%=14 (2%) Viral load in sex partner LWH at baseline: Undetectable viral load (<50 copies/mL), n (%): 754 (97%) Measured viral load: <200 copies/mL, n (%): 774 (99%) ≥200 copies/mL, n (%): 7 (<1%) Condom use: NR, only condomless acts were included in the analysis Use of HIV PrEP in HIV-negative partner: data for participants exposed to PrEP were removed from the analyses Comparator: None	Outcomes: Rate of phylogenetically linked HIV infections. (number of linked HIV infections/couple years of follow-up) Follow-up: 1,593 couple years Median follow-up/couple=2 years (IQR 1.1, 3.5 years) HIV negative partner: HIV testing baseline and every 6–12 months Sex partner LWH: Viral load tested baseline and every 6–12 months
Nyombayire *et al.*, 2021 ((16)) Rwanda	Prospective cohort Setting: Government clinics in Kigali Study period: 2010–2014	Heterosexual HIV-serodiscordant couples/sex partners Number of couples recruited n=3,777 Baseline characteristics: Number of couples with male sex partners LWH (M+/F-) n=1,947 Number of couples with female sex partners LWH (M-/F+) n=1,830 Age by sex overall, mean (SD), years: Male=35.3 (9.3) Female=29.6 (8.7) CD 4 of sex partners LWH mean (SD), (units NR)^b^ M+/F-=472.5 (234.6) M-/F+=525.4 (269.7) Couples with current ART use in sex partner LWH at baseline, n (%): 1,684 (44.6) M+/F- couples with no contraceptive/condom use, n (%): 640 (80.7%) M-/F+ couples with no contraceptive/condom use, n (%): 570 (76.8%)	Exposure: Sex partner LWH receiving ART ART regimen: NR ART adherence: NR Viral load in sex partner LWH across follow-up: NR Duration of ART in sex partners LWH at baseline, mean (SD) years 3.1 (2.3) Use of HIV PrEP in HIV-negative partner: NR Comparator: Sex partner LWH not receiving ART	Outcomes: HIV seroconversion in the HIV-negative partner; virological linkage analysis (for most but not all couples with seroconversion in the HIV-negative partner) Follow-up: Quarterly clinic visits for HIV-negative partners Median (SD) follow-up, years=1.4 (1.2)

Abbreviations: ART, antiretroviral therapy; CD 4, cluster of differentiation 4; CLAI, condomless anal intercourse; HIV, human immunodeficiency virus; IQR, interquartile range; LWH, living with HIV; M+/F-, male partner positive, female partner negative; M-/F+, male partner negative, female partner positive; NR, not reported; PEP, post-exposure prophylaxis; PrEP, pre-exposure prophylaxis; SD, standard deviation; STI, sexually transmitted infections

^a^ Adapted from the 2023 CADTH review

^b^ Data were available for 36% of sex partners LWH, only

**Table A3:** Risk of bias of new relevant studies to assess outcome of risk of HIV transmission^a^

Authors	Study participation	Study attrition	Prognostic factor measurement	Outcome measurement	Study confounding	Statistical analysis and reporting	Overall risk of bias
Bavinton *et al.*, 2018, ((17))	^b^	^c^	^b^	^b^	^c^	^b^	^c^
Rodger *et al.*, 2019, ((18))	^b^	^b^	^b^	^b^	^c^	^b^	^b^
Rodger *et al.*, 2016, ((19))	^b^	^b^	^b^	^b^	^c^	^b^	^b^
Nyombayire *et al.*, 2021, ((16))	^b^	^d^	^b^	^d^	^d^	^b^	^d^

Abbreviation: HIV, human immunodeficiency virus

^a^ To assess Risk of Bias, the Quality in Prognosis Studies (QUIPS) tool was used ((12)). It has six domains that critically appraise the validity and bias in included studies of prognostic factors. The domains are: study participation, study attrition, prognostic factor measurement, outcome measurement, study confounding, and statistical analysis and reporting

^b^ Low risk of bias

^c^ Moderate risk of bias

^d^ High risk of bias

**Table A4:** GRADE summary of findings^a,b^

Certainty assessment							Number of couples/person-years	Certainty of Evidence (GRADE)	Number of HIV transmission per 100 person-years (95% CI)
Number of studies	Study design	Risk of bias	Inconsistency	Indirectness	Imprecision	Publication bias			
**Outcomes: HIV incidence for unspecified sex acts (per person-years)**									
**Question 1: HIV incidence on ART^c^**									
1^d^ Cohort studies ((16))	Observational studies (cohort and cross-sectional)	Very serious^d^	Very serious^e^	Serious^f^	Serious^g^	Undetected	3,777/2,867.4	Viral load of the Partner LWH was not reported Use of ART by Partner LWH was self-reported, and levels of adherence could not otherwise be validated Very high loss to follow up (i.e. 35%) Study power was not addressed Very low certainty of evidence (◯◯◯◯^e,f,g^) Excluded	0.63 (0.38–1.00)

Abbreviations: ART, antiretroviral therapy; CI, confidence interval; GRADE, Grading of Recommendations Assessment, Development, and Evaluation; HIV, human immunodeficiency virus; LWH, living with HIV

Legend: ⨁⨁⨁⨁, high; ⨁⨁⨁◯, moderate; ⨁⨁◯◯, low; ⨁◯◯◯, very low

^a^ Setting: Community

^b^ Participants: Heterosexual

^c^ Viral load could be any level (fewer than or more than 200 copies/ml)

^d^ High risk of bias

^e^ Downgraded for inconsistency because the viral load of partner living with HIV was not reported and use of ART by partner living with HIV was self-reported, and levels of adherence could not otherwise be validated

^f^ Indirectness considered as serious because the study did not consistently account for condom use

^g^ Imprecision: Total numbers did not meet the optimum sample size. Because of insufficient sample size and follow-up time (i.e. below 2,000 participants and 4,000 person-years), imprecision was rated as serious

**Table A5:** GRADE summary of findings^a,b^

Certainty assessment							Number of couples/person-years	Certainty of Evidence (GRADE)	Number of HIV transmission per 100 person-years (95% CI)
Number of studies	Study design	Risk of bias	Inconsistency	Indirectness	Imprecision	Publication bias			
**Outcomes: HIV incidence for unspecified sex acts (per person-years)**									
**Question 1: HIV incidence on ART^c^**									
1^d^ Cohort study (17)	Cohort	Not serious	Not serious	Not serious	Very serious^d^	Undetected	NR/5.8	◯◯◯◯^e^ Very low (Excluded)	0.00 (0.00–63.32)
**Question 2: HIV incidence on ART + viral load suppression + no condom use^f^**									
2^g^ Cohort studies ((17,18))	Cohort	Not serious^g^	Not serious	Not serious	Serious^h^	Undetected	1,125/1,825.2	⨁⨁⨁⨁^e,i^ High	0.00(0.00–0.11)

Abbreviations: ART, antiretroviral therapy; CI, confidence interval; GRADE, Grading of Recommendations Assessment, Development, and Evaluation; HIV, human immunodeficiency virus; NR, number of participants not reported

Legend: ⨁⨁⨁⨁, high; ⨁⨁⨁◯, moderate; ⨁⨁◯◯, low; ⨁◯◯◯, very low

^a^ Setting: Community

^b^ Participants: gbMSM

^c^ Viral load could be any level (fewer than or more than 200 copies/ml)

^d^ Rated down because of the wide confidence interval crossing

^e^ No downgrade for publication bias

^f^ Viral load is suppressed at <200 copies/ml

^g^ Risk of bias was assessed as low for one study and as moderate for the other. However, both studies reported consistent results

^h^ Imprecision: Total numbers do not meet the optimum sample size. Because sample size and follow-up time were insufficient (i.e. below 2,000 participants and 4,000 person-years), imprecision was rated as serious

^i^ Dose response gradient: there was a dose-response relationship between the viral load and the absolute risk of transmission (Baggaley *et al.*) ((8)), so rated up for a dose-response gradient
